# Sexual dimorphism and female advantage hypothesis in the gynomonoecious-gynodioecious *Dianthus plumarius* (Caryophyllaceae)

**DOI:** 10.1093/aobpla/plad084

**Published:** 2023-11-28

**Authors:** Sabrina S Gavini

**Affiliations:** Grupo de Ecología de la Polinización, Instituto de Investigaciones en Biodiversidad y Medio Ambiente (INIBIOMA), CONICET—Universidad Nacional del Comahue, Quintral 1250, 8400, San Carlos de Bariloche, Rio Negro, Argentina

**Keywords:** Bumblebees, *Dianthus plumarius*, dimorphism, flower size, geitonogamy, gynodioecy, inbreeding depression, nectar production, ovule number, rewards

## Abstract

To explain the co-existence and maintenance of females along with hermaphrodite plants, the female advantage hypothesis has been proposed where females should show greater fecundity compared to their conspecific hermaphrodites. On the other hand, greater attraction would be selected in the hermaphrodites to increase their male function, potentially leading to larger showier flowers, with more rewards. Here, I tested the sexual dimorphism trade-off hypothesis with the gynomonoecious-gynodioecious *Dianthus plumarius* (Caryophyllaceae), in the gardens of Bariloche (Patagonia, Argentina). I measured in female and hermaphrodite plants: flower size, nectar volume and concentration, flower lifespan, ovule production, seed number, seed set and seed weight. Additionally, bagging and pollen supplementation experiments were carried out to evaluate pollen limitation, probability of apomixis, if spontaneous autogamy is possible, and to examine the importance of pollen origin. I found that hermaphrodite flowers are more attractive, with larger-sized flowers and higher nectar volume, whereas female flowers compensate with longer lifespan of stigmatic receptivity and more concentrated nectar. Despite ovule number was lower in female flowers, these showed higher seed set and produced more and heavier seeds than hermaphrodites under open pollination. No evidence of apomixis was found in females, but spontaneous autogamy may occur in hermaphrodites. Hand-pollination experiments showed first that both flower types suffered pollen limitation, but it was higher on hermaphrodite flowers. Finally, despite self-compatibility, pollen origin is important because hand self-pollination decreases seed weight. These findings provide strong evidence in support of the mechanisms and underlying conditions that would allow the co-existence and maintenance of female and hermaphrodite individuals within populations.

## Teaser

My findings provide evidence in support of the mechanisms and underlying conditions that would allow the co-existence of female and hermaphrodite plants within populations. The enigma lies in why females are maintained amid competition with hermaphrodites that gain fitness through both female and male functions. First, a higher attraction would be selected in hermaphrodites to increase their male function; certainly, hermaphrodites of *Dianthus plumarius* showed larger-sized flowers with higher nectar availability. Second, a necessary condition is that females should exhibit a fertility advantage. It is confirmed the existence of this female fertility advantage in *D. plumarius* . Particularly, female flowers showed extended stigmatic lifespan, and more concentrated nectar, but more importantly, these yielded more and heavier seeds compared to their hermaphrodite counterparts.

## Introduction

In animal-pollinated plants, floral traits strongly affect species fitness through pollinator attraction. Numerous studies have assessed the relationship between pollination visitation and intraspecific floral trait variation such as flower morphology, reward quantity, quality and presentation schedules and/or timing of flowering, among others (e.g. Fenster *et al.* 2004; Tsuji *et al.* 2020; Devegili and Farji-Brener 2021; Essenberg 2021). Gynodioecy, the co-occurrence of female and hermaphrodite individuals within populations of a given species, was believed to be one of the rarest sexual polymorphisms in angiosperms (≪1 % of species, see Godin and Demyanova 2013). However, gynodioecy is phylogenetically widespread, it is estimated that occurs in approximately 7–10 % of the flowering plant families (Delannay 1978; McCauley and Olson 2008) or up to ~21 % according to Caruso *et al.* (2016), and is mostly associated with perennial herbs, temperate climate and entomophilous pollination (Godin and Demyanova 2013; Caruso *et al.* 2016).

As with many other floral polymorphisms, the appearance and maintenance of gynodioecy remains intriguing (Dufay and Billard 2012), despite the great number of theoretical and empirical studies triggered by this phenomenon in the last decades (McCauley and Bailey 2009). Specifically, it raises the question of how female individuals are maintained while competing with hermaphrodites that gain fitness through both male (seed siring) and female (seed set) functions. Theory predicts that the persistence of females, along with hermaphrodites, can only occur when there is a female advantage over hermaphrodites that allows female plants to achieve higher fecundity by producing more seeds and/or seeds of higher quality than hermaphrodites (Lewis 1941; Charlesworth and Charlesworth 1978). It is believed that the evolution of gynodioecy from hermaphroditism can involve changes in the floral structure such that male or female fitness is enhanced in hermaphrodite and female plants, respectively (Gibson and Diggle 1997). Ultimately, female flowers can have a much larger female function compared to hermaphrodites because they do not allocate resources to the male function and do not pay the cost of producing inbred seeds. Even though a female advantage should be needed in gynodioecious species, its magnitude is theoretically expected to vary with the genetic determination of the male sterility. In particular, the magnitude of the female advantage can be low (i.e. females are better, but not necessarily twice better, than hermaphrodites) when sex determination is nuclear-cytoplasmic, whereas at least a 2-fold advantage is required in the case of purely nuclear-transmitted male sterility (Lewis 1941; Shykoff *et al.* 2003; Dufay and Billard 2012). Previous meta-analyses showed evidence of this female advantage in many, but not all, gynodioecious species (reviewed in Shykoff *et al.* 2003; Dufay and Billard 2012; Varga 2021). On the other hand, hermaphrodites are generally more attractive to pollinators because they produce much larger flowers (Eckhart 1993, 1999; Williams *et al.* 2000; Shykoff *et al.* 2003; Asikainen and Mutikainen 2005; McCall and Barr 2012; Miyake *et al.* 2018) and provide greater diversity and availability of rewards than female flowers (Eckhart 1999; Ashman *et al.* 2000). As a result, in gynodioecious species, pollinators are expected to visit the larger, more conspicuous hermaphrodite flowers first to facilitate the pollination of the expected less showy and strictly outcrossing female ones (Darwin 1877).

**Figure 1.**
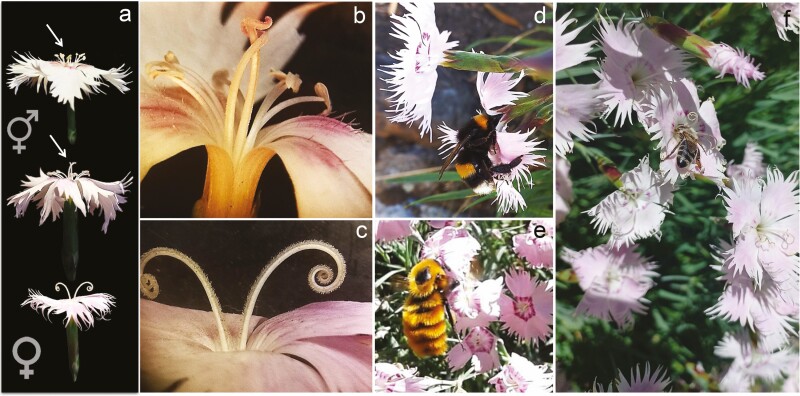
Flowers of *D. plumarius*. (A) Hermaphrodite flower (top and middle) and female flower below, the arrow on the top flower points the anthers and the arrow on the middle flower points the developing stigma. (B) Detail of the hermaphrodite flower, showing the anthers and the curly two stigmas close to elongating. (C) Detail of female flower with the two separate stigmas with a long adaxial stigmatic surface with a big curl at the end. (D) Frequent daytime pollinator in the region, the exotic and invasive bumble bee *Bombus terrestris* (Apidae) drinking nectar from a female flower. (E) Diurnal pollinator, nowadays less frequent to observe, the native bumble bee *Bombus dahlbomii*. (F) *Apis melifera* honey bee on female flowers, which are frequent diurnal pollinators.

A lineage with wide-ranging sexual polymorphisms is the carnation family (Caryophyllaceae) (Jürgens *et al.* 2002; Casimiro-Soriguer *et al.* 2015). Many species of *Silene* and *Dianthus* have been used as model systems for understanding the evolution of gynodioecious or gynomonoecious-gynodioecious mating systems (e.g. Collin and Shykoff 2003; Shykoff *et al.* 2003; Lafuma and Maurice 2006; Bernasconi *et al.* 2009; Casimiro-Soriguer *et al.* 2015). In the case of *Dianthus*, many aspects of the sexual dimorphism were assessed in *D. sylvestris* (Collin *et al.* 2002; Collin and Shykoff 2003), *D. shinanensis* (Kuhara and Sugawara 2002), *D. superbus* (Sugawara *et al.* 1994; Kuhara and Sugawara 2002; Miyake *et al.* 2018) and *D. pavonius* (Bruns *et al.* 2018), all in the species native ranges (Europe or Asia). However, to date, there is no clear evidence in favour of the female advantage hypothesis for this genus. For instance, in *D. sylvestris*, despite seed mass being higher in females, there were no differences in seed number and germination between morphs (Collin *et al.* 2002). In *D. shinanensis*, hermaphrodites showed better female success, whereas in *D. superbus* female success was either similar between morphs (Kuhara and Sugawara 2002) or higher in hermaphrodites (Sugawara *et al.* 1994). Consequently, the purpose of the present study was to explore the reproductive nature of *Dianthus plumarius* (Fig. 1), a gynomonoecious-gynodioecious species native to central Europe. In the context of this study, there are no wild populations, *D. plumarius* is exotic and is only cultivated as an ornamental in the gardens of San Carlos de Bariloche in Patagonia, Argentina. Using *D. plumarius* as a model system, I tested the previously explained sexual dimorphism trade-off hypothesis of hermaphrodite flowers being more attractive than female flowers, but female ones showing an advantage over hermaphrodites in terms of higher female reproductive success. I measured in female and hermaphrodite plants; flower size, floral rewards and flower lifespan. Additionally, bagging experiments and pollen supplementation experiments were carried out to evaluate pollen limitation, the probability of apomixis, whether spontaneous autogamy is possible, and to examine the importance of pollen origin. Ultimately, I compared the reproductive success of female and hermaphrodite flowers. Accordingly, I predicted larger flowers and reward production in hermaphrodites, whereas a higher number of seeds and/or seed quality were expected in females.

## Materials and Methods

### Study species and site

*Dianthus* (Caryophyllaceae) comprises more than 300 species and occurs in temperate regions worldwide. *D. plumarius*, better known as cottage or feathered pink and characterized by its feathery-edged petals (Fig. 1A), is an entomophilous long-lived perennial herb native of Europe. Its flowers have five petals and are fragrant, pink or alternatively fully white, sometimes with red/burgundy bands surrounding the centre, and they produce nectar from the base of a narrow calyx tube. Individual plants may be large and with many flowers, sometimes having >100 flowers opening simultaneously, providing hermaphrodites the opportunity for selfing. *Dianthus* species are self-compatible; however, selfing can only occur through geitonogamy because flowers are strongly protandrous. Hence, hermaphrodite flowers produce pollen first, developing a receptive stigma after all the anthers withered (Fig. 1A and B). Instead, the stigma of female flowers is receptive as soon as the flower opens (Fig. 1A and C). Anthers are still present on female flowers but they are extremely reduced and not functional. Both types of flowers wither following pollination. According to previous studies with other *Dianthus* species, most common pollinators are moths, butterflies and bees (Jürgens *et al.* 2002, 2003; Kuhara and Sugawara 2002). Field observations indicate that *D. plumarious* is gynomonoecious-gynodioecious, that is, populations composed of hermaphrodite individuals with only perfect flowers, female individuals with only pistillate flowers and gynomonoecious individuals with both perfect and pistillate flowers.

The study was performed in the city of San Carlos de Bariloche (41°08ʹS, 71°18ʹW), located in the northwest of Patagonia Argentina (Río Negro province) and included within the limits of the Nahuel Huapi National Park. Climate is temperate-cold and humid with a Mediterranean-type rainfall regime with rain and snowfall mainly during winter (Dimitri 1972). The flowering of *D. plumarius* ranges from late spring to the early summer. The study was conducted from November 2022 to January 2023. Different streets of the urban area and neighbourhoods were visited, and if present, *D. plumarius* plants were marked. All flowers were inspected almost daily to determine the plant’s floral morph. From this survey, I found a total of 69 plants; 32 hermaphrodites, 22 females and 15 gynomonoecious individuals, scattered in 8 sites.

### Morphological, phenological and nectar differences between flower morphs

I recorded the total number of flowers produced per plant in all 69 plants, and the type of individual (female, hermaphrodite, gynomonoecious) from daily monitoring as some flowers withered and new flowers developed. In the case of gynomonoecious individuals, the number of female and hermaphrodite flowers was counted to determine if there was a predominant morph. To determine morphometric differences between hermaphrodite and female flowers I collected 160 flowers from 40 individual plants (20 female plants vs. 20 hermaphrodite plants, 80 female flowers vs. 80 hermaphrodite flowers), gathering 4 flowers per plant, and measured corolla diameter, petal length and calyx tube length using callipers. In addition, I labelled flower buds that were inspected twice a day, every day that the flower lasted to examine the flowers’ lifespan. Specifically, I followed 12 females and 22 hermaphrodite flowers since the bud stage.

I measured nectar volume with 1 µL capillaries and nectar concentration using a hand-held refractometer (°Brix). Nectar measurements were performed in unbagged flowers, that is, flowers exposed to visitors, in order to assess natural levels of nectar available, and in bagged flowers, that is, which were bagged from the bud stage, to assess the levels of nectar that each flower morph can produce. For unbagged flowers, I measured nectar volume in 316 flowers from 50 individual plants (22 female plants vs. 28 hermaphrodite plants, 162 female flowers vs. 154 hermaphrodite flowers) by gathering 5–8 flowers per plant. About 20 flowers were empty (11 female flowers and 9 hermaphrodite flowers), thus analyses of nectar volume comprised 296 flowers only. From these 296 flowers, I could measure nectar concentration in 251 flowers (22 female plants vs. 28 hermaphrodite plants, 121 female flowers vs. 130 hermaphrodite flowers) with 4–6 flowers per plant, because the amount of nectar extracted was too low in 45 flowers. I bagged a total of 130 flower buds, 5 buds per plant in 26 plants (13 female plants vs. 13 hermaphrodite plants, 65 female flowers vs. 65 hermaphrodite flowers). I was able to measure nectar volume and concentration for all 130 flowers.

### Natural and manual pollination experiments

To measure natural levels of seed production in both morphs, several flower buds per plant were randomly selected, labelled and covered with mesh-net bags. Bagging and pollen supplementation experiments were carried out to evaluate pollen limitation, probability of apomixis, whether spontaneous autogamy is possible despite protandry, and to examine the importance of pollen origin (self vs. cross). In the case of hermaphrodite flowers, one of four treatments was applied: (i) bagged flowers were left untouched [*n* = 50 flowers] to test for spontaneous autogamy, (ii) open pollination with flowers exposed and eventually visited by pollinators [*n* = 200], (iii) hand self-pollination (i.e. hand-pollinated flowers with pollen of a male-phase flower from the same individual plant) [*n* = 66] or (iv) hand cross-pollination (i.e. hand-pollinated flowers with pollen from a plant, at least, 10 m apart) [*n* = 66]. Flowers in treatments (iii) and (iv) were re-bagged after hand pollination for a month until fruit maturation. In the case of female flowers, one of three treatments was applied: (i) bagged flowers left untouched [*n* = 50] to test for apomixis (i.e. asexual reproduction without fertilization leading to the formation of seeds), (ii) open pollination with flowers exposed and eventually visited by pollinators [*n* = 125] or (iii) hand cross-pollination [*n* = 70]. Experimental flowers [*n* = 627] ­corresponded to 28 plants (11 female plants vs. 17 hermaphrodite plants), with pollination treatments replicated several times within plants. After fruit collection, I counted the number of developed and aborted seeds. The sum of both seed classes was considered as an estimated of total number of ovules per flower. As a measure of seed quality, developed seeds were weighed using an analytical balance. I weighed 5457 seeds split across *n* = 200 samples, corresponding to the following 5 treatments: open-pollinated female flowers [*n* = 40 samples], cross-pollinated female flowers [*n* = 40], open-pollinated hermaphrodite flowers [*n* = 40], cross-pollinated hermaphrodite flowers [*n* = 40] and self-pollinated hermaphrodite flowers [*n* = 40]. Each sample is a random subset of seeds from an experimental fruit. Seed samples corresponded to 18 plants (8 female plants vs. 10 hermaphrodite plants), from 4 to 5 flowers per treatment per plant (i.e. 10 random flowers per female plant and 12 random flowers per hermaphrodite plant). As an estimation of individual seed weight, the following formula was applied: *individual seed weight* = *total weight of seeds in the sample* / *total number of seeds in the sample*.

### Statistical analyses

Analyses were conducted in R Software version 4.3.0 (R Development Core Team 2023). General and generalized linear mixed-effect models were used with the glmmTMB package (Brooks *et al.* 2017). The number of flowers per plant was analysed with plant morpho-type (female, hermaphrodite, gynomonoecious) as a fixed effect and site as a random effect, considering a negative binomial error distribution and a log-link function. All subsequent analyses include the comparison between female and hermaphrodite plants, that is, gynomonoecious individuals were discarded, because of the scarcity of plants of this morpho-type and the large differences in the flower type ratio within plants (see results). The three morphometric traits (corolla diameter, petal length, calyx tube length) were analysed with flower type (hermaphrodite vs. female) as a fixed effect and plant individual as a random effect, considering a normal distribution and identity-link function. I analysed the number of ovules (poisson error distribution), nectar volume (gamma error distribution) and nectar concentration (gaussian error distribution), following the same model structure as before. From the pollination experiment, experimental flowers from spontaneous autogamy [hermaphrodite flowers, *n* = 50] and apomixis [female flower, *n* = 50] treatments were not included in the statistical analyses because of the lack of fruits (see ‘Results’ section). I analysed fruit data only from experiments of cross-pollinated, open-pollinated and self-pollinated flowers [*n* = 527]. In female plants, there were only two levels of the pollination treatment (cross pollination, open pollination), whereas in hermaphrodite plants there were three (cross pollination, open pollination, self pollination). Because this disabled the possibility of assessing the interaction between the pollination treatment and the flower morph, I decided to perform models for each flower morph separately [*n* = 332 of hermaphrodite experimental flowers, *n* = 195 of female experimental flowers]. Accordingly, I used the pollination treatment as a fixed effect and plant individual as a random effect, to analyse the number of seeds per fruit (negative binomial error distribution), the ratio of formed seeds per fruit or seed set (beta-binomial error distribution) and seed weight (gamma error distribution in female-flower model, gaussian error distribution in hermaphrodite-flower model). Finally, the magnitude of the female advantage was quantified as the ratio of the average value in females divided by the average value in hermaphrodites. For this, I first used data from open-pollinated flowers in order to assess whether females benefit from a female advantage or disadvantage in natural conditions (Dufay and Billard 2012), secondly, I used data from hand cross-pollinated flowers. For each individual plant, I performed the following calculation: *number of flowers produced × seed number per fruit*, and *number of flowers produced × seed weight*. I calculated the average value for both sexual morphs and then the ratio. A ratio > 1 would imply a female advantage in females, if the ratio = 1 there is no advantage, a ratio < 1 would imply a female disadvantage compared to hermaphrodites.

## Results

### Morphological, phenological and nectar differences between flower morphs

Among the 69 plants, the number of flowers varied considerably, with several plants with >100 flowers, in fact, one female plant had about 800 flowers. There was no evidence that the total number of flowers differed between plant sexual morpho-types (*χ*² = 0.819, df = 2, *P* = 0.664). Full-hermaphrodite plants had 89.6 ± 25.1 flowers, full-female plants 117.4 ± 36.5, whereas gynomonoecious plants had 109.8 ± 37.0 flowers (estimated mean ± 1 SE). On average, ~41 % of the flowers are simultaneously open. In the 15 gynomonoecious plants, 3 possible scenarios were found: female flowers prevail (*n* = 6 plants), hermaphrodite flowers prevail (*n* = 6) or both morphs were similarly represented (*n* = 3). I considered it as a predominant floral morph when one type of flower represented >65 % of the total number of flowers present in the plant. Examples of extreme cases where one floral morph dominated, and could have been erroneously categorized as a full-hermaphrodite individual, occurred in two plants where the female flowers represented <2 % of the total number of flowers (1.1 % and 1.8 % of all flowers were female).

**Figure 2.**
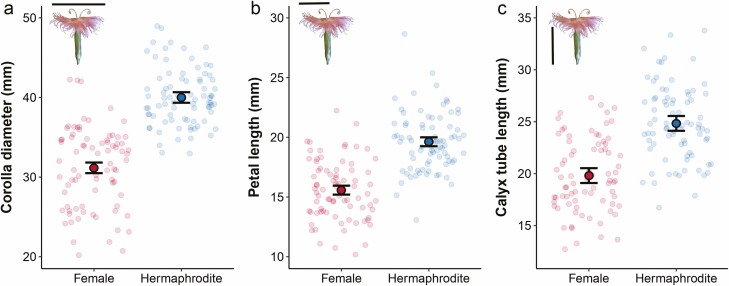
Morphometric measurements in female and hermaphrodite flowers. Observed data and models estimated means (± 1SE, in mm) for (A) corolla diameter, (B) petal length and (C) calyx tube length.

All measurements associated with flower size (corolla diameter, petal length, calyx tube length) strongly evidenced that hermaphrodite flowers were larger than female flowers (Fig. 2A corolla diameter: *χ*² = 88.4, df = 1, *P* < 0.0001; Fig. 2B petal length: *χ*² = 58.6, df = 1, *P* < 0.0001; Fig. 2C calyx tube length: *χ*² = 24.7, df = 1, *P* < 0.0001). Using fruits collected from the pollination experiments (*n* = 527, 195 female flowers vs. 332 hermaphrodite flowers), I compared ovule numbers between floral morphs. Hermaphrodite flowers produced 13 % more ovules than female flowers (*χ*² = 29.9, df = 1, *P* < 0.0001) with hermaphrodites having 69.8 ovules/flower [lower CI 68.0 – upper CI 71.7] whereas females showed 61.9 ovules/flower [lower CI 59.9 – upper CI 64.0].

In all locations, I observed that the main plant sexual morpho-types (female and hermaphrodite) began to flower together. However, towards the end of the flowering season of *D. plumarius*, the plants still in flower corresponded to hermaphrodite individuals only. Flowers remain open during the day and night. Female flowers, after open, lasted (mean ± 1 SE) 8.5 ± 0.9 days, ranging from 5 to 13 days. In the case of hermaphrodite flowers, I recorded the number of days in the male phase, intermediate phase (i.e. stigma present but not fully developed and, almost, all anthers withered), and in the female phase. Number of days in the male phase was 2.6 ± 0.2 (range from 1 to 4 days). Number of days in the intermediate phase was 0.7 ± 0.1 (range from 0 to 1 day). These values are due to the fact that, in 7 of the 22 flowers, the previous night was in the male phase and the next early morning it had a fully developed stigma, and thus they were recorded as in the female phase. Finally, the number of days in the female phase was 4.1 ± 0.4 (range from 2 to 9 days). Accordingly, the total number of days that a hermaphrodite flower lasts was 7.4 ± 0.4, ranging from 5 to 12 days. The hermaphrodite flower with the shortest lifespan presented 2 days in the male phase and 3 days in the female phase. Instead, the longest-lived hermaphrodite flower was 2 days in the male phase, 1 day in the intermediate phase and 9 days in the female phase. Therefore, flower lifespan is mainly determined by the length of the female phase, which, in turn, is determined by pollination success. In fact, hand-pollinated flowers or flowers visited by insects closed the next day, and showed shrivelled stigmas. Ultimately, the receptivity lifespan of female flowers was 2.1 longer than those of the hermaphrodites.

**Figure 3.**
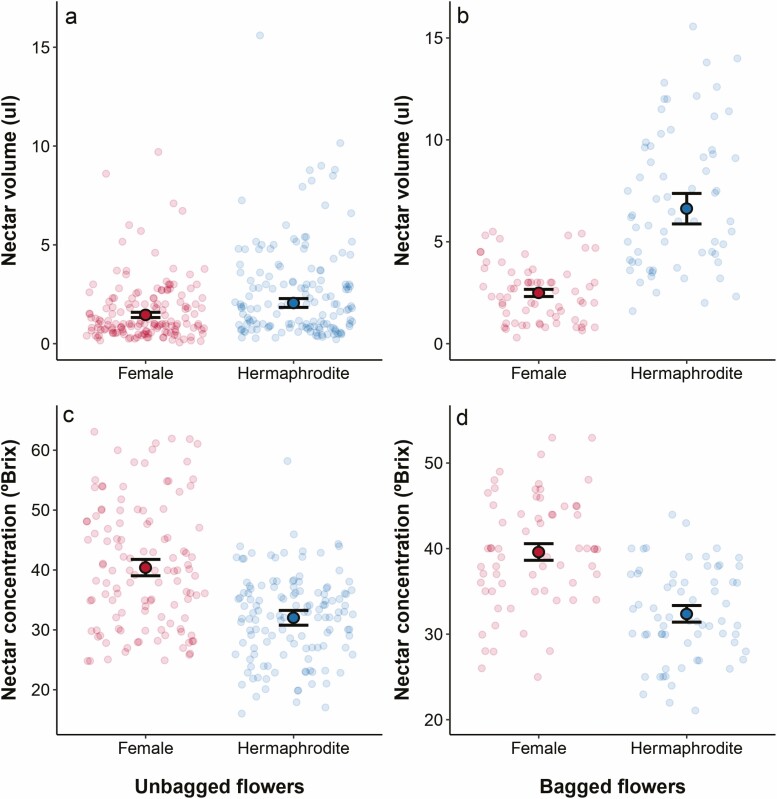
Nectar measurements in female and hermaphrodite bagged and unbaggaed flowers. It is shown the observed data and models estimated means (± 1 SE) for nectar volume (µL) in (A) unbagged and (B) bagged flowers, and nectar concentration (ºBrix) in (C) unbagged and (D) bagged flowers, as a function of whether the flowers are female or hermaphrodite.

Measurements in unbagged flowers evidence that nectar volume is 41 % higher in hermaphrodite flowers (Fig. 3A, *χ*² = 6.99, df = 1, *P* = 0.008211). In female exposed flowers, nectar volume ranged from <1 to 9.7 µL and in hermaphrodite flowers from <1 to 15.6 µL (Fig. 3A). The difference between floral morphs in nectar volume became much stronger in bagged flowers (*χ*² = 60.79, df = 1, *P* < 0.0001, Fig. 3B). By comparing unbagged and bagged flowers, nectar volume increased 1.7 times in female flowers and 3.2 times in hermaphrodite flowers. On the other hand, nectar concentration (ºBrix) was, on average, 25 % higher in female flowers (Fig. 3C and D), a pattern that persisted regardless of whether flowers were unbagged (*χ*² = 20.73, df = 1, *P* < 0.0001) or bagged (*χ*² = 27.70, df = 1, *P* < 0.0001).

### Natural and manual pollination experiments

**Figure 4.**
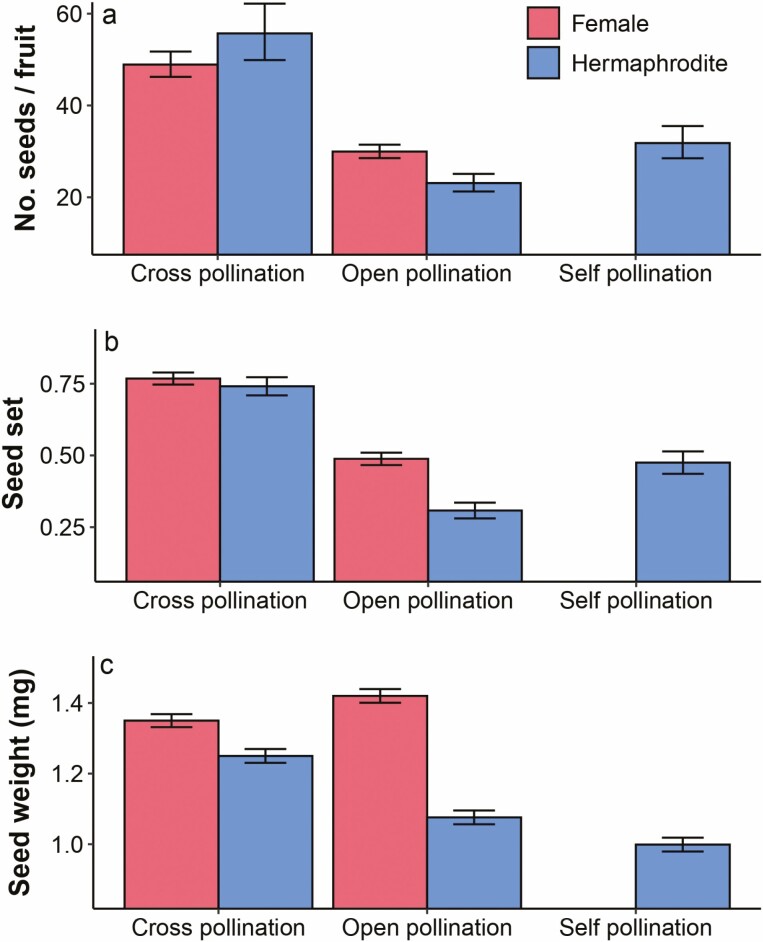
Reproductive success in female and hermaphrodite flowers subjected to different pollination treatments. It is shown models estimated means (± 1SE) of (A) number of seeds/fruit, (B) seed set and (C) seed weight (mg) of female cross- and open-pollinated flowers and hermaphrodite cross-, open- and self-pollinated flowers.

None of the bagged female flowers produced fruit and seeds, thus no apomixis occurred in the female flowers of *D. plumarius*. In the case of bagged hermaphrodite flowers, only eight flowers formed fruit, thus showing an autogamous fruit set of 16 %. Instead, all open-pollinated (control) and hand-pollination treatments in both floral morphs formed fruits. Artificial pollination experiments evidenced pollen limitation in both floral morphs (Fig. 4), because hand-pollinated flowers produced a higher number of seeds and seed set than open-pollinated flowers (Table 1). Overall, under open pollination female flowers showed greater female success than hermaphrodite flowers (Fig. 4), specifically female flowers exposed to pollinators produced 29 % more seeds and showed 58 % higher seed set than hermaphrodite flowers.

**Table 1.** Results of the pollination experiments on reproductive success. Generalized mixed-effect models statistics showing the effect of the pollination treatment on number of seeds per fruit, ratio of formed seeds (seed set), seed weight (mg) in female and hermaphrodite flowers separately. The pollination treatment in female flowers has only two levels (cross pollination and open pollination or control), in hermaphrodite flowers has three levels (cross pollination, open pollination or control and self pollination).

	Female flowers Cross pollination vs. Open pollination		Hermaphrodite flowers Cross-pollination vs. Open pollination vs. Self-pollination	
	*χ*² _(1)_	*P*	*χ*² _(2)_	*P*
No. seeds/fruit	80.77	<0.0001	71.75	<0.0001
Seed set	100.74	<0.0001	177.85	<0.0001
Seed weight (mg)	7.91	0.00491	88.09	<0.0001

In the case of females, cross-pollinated flowers produced 64 % more seeds (Fig. 4A) and showed 57 % higher seed set (Fig. 4B) than open-pollinated flowers (Table 1). In the case of hermaphrodites, the lowest reproductive success was found in open-pollinated flowers (Fig. 4 and Table 1), evidencing greater pollen limitation in hermaphrodite than in female flowers. Additionally, there were differences between hand-pollination treatments (cross pollination vs. self pollination: *z*.ratio = 4.71, *P* < 0.0001 for seed number; *z*.ratio = 7.35, *P* < 0.0001 for seed set). Whereas hand self-pollination resulted in 37 % more seeds and 54 % higher seed set than open-pollinated flowers, hand cross-pollination resulted in increases of ~140 % for both seed number and seed set compared to open-pollinated flowers (Fig. 4).

Seeds from female flowers were, on average, 32 % heavier than seeds from hermaphrodite flowers under open pollination (Fig. 4C). Instead, under the cross-pollination treatment, seeds from females were only 8–9 % heavier than the seeds from hermaphrodites. Moreover, seed weight of females, surprisingly, was 5.2 % higher in the open-pollination than cross-pollination treatment (Table 1 and Fig. 4C). Specifically, open-pollinated female flowers produced seeds weighing, on average, 1.42 mg. Contrariwise; seed weight in hermaphrodites was 16 % higher in the cross-pollination treatment compared to open-pollination (Table 1). There were also differences between open-pollination and self-pollination treatments (*z*.ratio = 2.79, *P* = 0.0167). The self-pollination treatment represented the lowest seed quality scenario with seeds weighing, on average, a little less than 1 mg (Fig. 4C).

Finally, for open-pollinated data females showed a female advantage in both reproductive traits explored (female/hermaphrodite ratio = 1.70 for *number of flowers produced × seed number per fruit*; ratio = 1.73 for *number of flowers produced* × *seed weight*). In the case of hand-pollinated data, ratios decreased but were still greater than 1 (ratio = 1.16 for *number of flowers produced × seed number per fruit*; ratio = 1.42 for *number of flowers produced* × *seed weight*).

## Discussion

A necessary condition for the appearance and maintenance of females in gynodioecious species is that a female fertility advantage occurs. In this study, I tested this hypothesis in the gynomonoecious-gynodioecious pink *D. plumarius*. Results provide strong evidence in support of the female-advantage hypothesis. First, I found that hermaphrodite flowers are indeed more attractive, with larger-sized flowers and higher nectar availability. Female flowers compensate for their reduced attractiveness with longer stigmatic receptivity lifespan and, curiously, more concentrated nectar. Second, although no apomixis occurs in the obligate outcrossing females, according to pollination experiments they showed greater female success than hermaphrodites. Female flowers produced more seeds per fruit, higher seed set and heavier seeds; therefore, presumably of better quality, than hermaphrodites. Even though hermaphrodite flowers are strongly protandrous, spontaneous autogamy may occur. Moreover, geitonogamy can occur very easily owed to the profuse number of flowers simultaneously open in each plant. Also, hand-pollination experiments indicate that pollen origin is important. Particularly, despite self-pollination translated into higher seed number and seed set than open pollination, seed weight declined. Overall, the study provides strong support for the mechanisms and underlying conditions that would lead to the co-existence of females along with hermaphrodite plant individuals.

Flowering plants display a wide diversity of floral traits that are hypothesized to evolve in response to natural selection by pollinators (Harder and Johnson 2009). Selection should favour traits that improve pollen transfer and fertilization success. In the case of gynodioecy, this should lead to sexual dimorphisms in pollinator-attracting traits, rendering hermaphrodite flowers being more attractive to guarantee pollen transport and pollination success of the strictly outcrossing females. In particular, flower size is expected to play a key role in attracting pollinators (Goodwillie *et al.* 2010). Sexual dimorphism in flower size has been previously studied in several species of *Dianthus*. Results of this study show that in *D. plumarius* the hermaphrodite flowers were larger than females, a pattern consistent with *D. sylvestris* (Collin *et al.* 2002; Collin and Shykoff 2003), *D. shinanensis* (Kuhara and Sugawara 2002), *D. superbus* (Kuhara and Sugawara 2002; Miyake *et al.* 2018) and *D. pavonius* (Bruns *et al.* 2018). Ultimately, my findings add to previous reviews showing larger hermaphrodite flowers to be a common phenomenon in gynodioecious plant systems (Eckhart 1999; Shykoff *et al.* 2003). Pollinators may select to visit the larger flowers because they are more conspicuous and/or because they may be associated with larger rewards too (Blarer *et al.* 2002). Indeed, the hermaphrodite flowers of *D. plumarius* were the ones containing higher nectar volume, with 20 % of the bagged flowers presenting, at least, 10µL.

On the other hand, female flowers, reliant on the presence and abundance of hermaphrodites for pollen availability, compensate their supposedly lower attractiveness (i.e. smaller flowers and less nectar volume) with twice longer stigmatic receptivity lifespan than hermaphrodites. An unexpected result was consistently a more concentrated nectar in these flowers. High sugar concentrations offer greater energetic reward for bees, however as concentration increases so does the viscosity (Nardone *et al.* 2013), which makes it harder for bees to imbibe with their tongues (Kim *et al.* 2011). Consequently, pollinators may spend more time on flowers with higher nectar concentrations. This increased time per flower could benefit female flowers if increased flower handling times are associated with increased pollen deposition. A previous study experimentally showed that the higher the nectar concentration, the longer the visit per flower (Thomson *et al.* 2012). Specifically, in flowers with a concentration of 30 %, bumblebee visits lasted 5–6 s, whereas visits on flowers with a concentration of 10 % lasted 1–2 s only. Their findings also showed that flowers with concentrated nectar receive more pollen than those with dilute nectar (Thomson *et al.* 2012). As follows, more concentrated nectar could be a side strategy of the female flowers to enhance the time spent of pollinators and, hence, their pollination success.

Females are, supposedly, at a selective disadvantage because they contribute genes to the next generation through ovules only, whereas hermaphrodite flowers through ovules and pollen. Consequently, for females to be maintained in gynodioecious populations they must have some compensating selective advantage, specifically through better seed production and/or quality compared with hermaphrodites (Dufay and Billard 2012; Varga 2021). Here, I found evidence in support of the female-advantage hypothesis. First, under open pollination, the female flowers of *D. plumarius* showed higher seed production per fruit and seed set than hermaphrodite flowers. Second, fruits from females formed heavier seeds, so will possibly have an advantage over small seeds in total nutrient content. Third, comparisons between hand-pollinated flowers and flowers exposed to pollinators indicated that both floral types suffer pollen limitation, but it is lower in females. Finally, the magnitude of the female advantage was estimated. Females of *D. plumarius* showed a female advantage magnitude of ~1.7 for open-pollinated flowers. Curiously, a relatively similar value was reported for *Dianthus sylvestris* (1.62), according to the estimations by Dufay and Billard (2012) for the same reproductive trait. Although the genetic system underlying male sterility is known for a very limited number of species, the low female advantage value found here (greater than 1 but lower than 2) agrees with a nuclear-cytoplasmic sex determination, where females are better, but not necessarily twice better, than hermaphrodite individuals. Consistently with these findings, recent studies have determined cytoplasmic male sterility in *Dianthus spiculifolius* (Liu *et al.* 2023). The next step could be assessing seed germination success and seedling’s growth rate, to confirm that female plants would have a higher contribution or probability to succeed in the following generation.

The female advantage observed in *D. plumarius* may result from a combination of several non-exclusive mechanisms, such as (i) re-allocation of resources saved from pollen production (Shykoff *et al.* 2003), (ii) higher style receptivity and/or (iii) better ovule quality. For instance, the style of females may be more efficient for the capture and attachment of pollen; as seen in *Silene acaulis*, where the female flowers had longer styles and longer stigma papillae, hence larger stigmatic surfaces, than hermaphrodite flowers (Shykoff 1992). On the other hand, ovule production in *Dianthus* seems to vary considerably within and between species; for example, the number of ovules per flower in *Dianthus superbus* was not different between hermaphrodites and females, while in *D. shinanensis* hermaphrodites produced more ovules (Kuhara and Sugawara 2002). Here, consistent with the findings in *D. shinanensis*, I found that hermaphrodite flowers of *D. plumarius* produce a higher number of ovules than females. Accordingly, a trade-off between ovule quantity and quality might exist, so that lower ovule quantity in females may underlie better quality per ovule. Furthermore, since apomixis does not occur, (iv) females’ progeny results exclusively from obligate outcrossing. This selfing avoidance enhances seed quality, in comparison with the self-compatible hermaphrodite flowers whose progeny can also result from self-pollination, and thus are less suitable due to inbreeding depression. In fact, under natural conditions, hermaphrodites of the gynodioecious *Fragaria vesca* produce seeds by self-fertilization 75 % of the times (Li *et al.* 2011). Despite strong protandry, hermaphrodites of *D. plumarius* can suffer spontaneous autogamy as suggested by bagging experiments, but the odds are low. Yet, since numerous flowers are simultaneously open per plant, self-pollination can easily occur through geitonogamy (Williams *et al.* 2000). Geitonogamy in hermaphrodites settles the grounds for the female fitness-advantage if such selfing results in inbreeding depression (Eckert 2000).

Hand self-pollination experiments in hermaphrodite flowers led to higher seed numbers and seed set compared to flowers exposed to pollinators but were lower than cross-pollinated flowers. Autogamy or geitonogamy may provide reproductive assurance in hermaphrodite plants of *D. plumarius* when pollination services and/or hermaphrodite neighbour plants are scarce. However, the decrease in the weight of seeds under the self-pollination treatment can be viewed as evidence of early inbreeding depression. Seeds from self-pollinated flowers weighed, on average, <1 mg; therefore, their germination and vigor may be compromised (Massimi 2018). A reduction in seed quality that resulted from selfing in hermaphrodites is consistent with the findings reported for other gynodioecious species such as *Silene nutans* (Dufay *et al.* 2010), *Dianthus sylvestris* (Collin *et al.* 2009) and *Dianthus pavonius* (Bruns *et al.* 2018). Pollen donor quality may also explain unexpected results in female flowers. As predicted, the reproductive success of female flowers increased under the cross-pollination treatment. However, seed weight was found to be better under open pollination. A possible explanation for this is that for the cross-pollination treatment, I provided fresh pollen from only one distant donor within the site, whereas it is possible that more distant pollen and/or greater donor diversity arrived to the stigmas of the flowers exposed to pollinators. Flower stigmas can be viewed as the arena in which prezygotic mate selection occurs (Aizen *et al.* 1990; Niesenbaum 1999; Erbar 2003). Under more competitive conditions particular pollen grains develop vigorous pollen tubes, increasing the probability that a superior male gametophyte achieves fertilization (Niesenbaum 1999; Paschke *et al.* 2002), potentially resulting in better-quality embryos. Accordingly, open pollination flowers may increase female offspring fitness only if post-pollination selection favours competitively superior, more spatially distant, genetically more diverse, or less closely related pollen (Aizen *et al.* 1990; Paschke *et al.* 2002; Souto *et al.* 2002) compared with the single-donor pollen sample I provided under the cross-pollination treatment.

In conclusion, these findings strongly support the existence of a female advantage in *D. plumarius*. Overall, results suggest that inbreeding depression following selfing in the attractive hermaphrodite flowers likely contributes to the maintenance of female plants. But this effect is also accompanied by significant features shown by female flowers such as selfing avoidance, twice longer stigmatic receptivity lifespans and more concentrated nectar, which may act as a side strategy to increase the time spent by pollinators during nectar collection, thus, enhancing pollination success. All this has potentially contributed to the female advantage reported here for all measured traits (higher seed number, greater seed set, higher seed weight) over hermaphrodites, and explains the co-existence and maintenance of females along with hermaphrodite plants.

## Data Availability

Data deposited at Zenodo: https://zenodo.org/doi/10.5281/zenodo.10073115
